# The role of physical activity and heart rate variability for the control of work related stress

**DOI:** 10.3389/fphys.2014.00067

**Published:** 2014-02-21

**Authors:** Laís Tonello, Fábio B. Rodrigues, Jeniffer W. S. Souza, Carmen S. G. Campbell, Anthony S. Leicht, Daniel A. Boullosa

**Affiliations:** ^1^Department of Physical Education, Catholic University of BrasiliaBrasilia, Brazil; ^2^Institute of Sport and Exercise Science, James Cook UniversityQueensland, QLD, Australia

**Keywords:** autonomic nervous system, physical fitness, exercise, allostatic load, employees

## Abstract

Physical activity (PA) and exercise are often used as tools to reduce stress and therefore the risk for developing cardiovascular diseases (CVD). Meanwhile, heart rate variability (HRV) has been utilized to assess both stress and PA or exercise influences. The objective of the present review was to examine the current literature in regards to workplace stress, PA/exercise and HRV to encourage further studies. We considered original articles from known databases (PubMed, ISI Web of Knowledge) over the last 10 years that examined these important factors. A total of seven studies were identified with workplace stress strongly associated with reduced HRV in workers. Longitudinal workplace PA interventions may provide a means to improve worker stress levels and potentially cardiovascular risk with mechanisms still to be clarified. Future studies are recommended to identify the impact of PA, exercise, and fitness on stress levels and HRV in workers and their subsequent influence on cardiovascular health.

## Introduction

The adaptive process by which an organism maintains homeostasis is known as allostasis with variable allostatic loads commonly experienced by humans (McEwen and Seeman, 1999; Frodl and O'Keane, 2012). When exposure to chronic stress becomes excessive, the allostatic load experienced may promote important alterations in stress sensitive systems that are intimately linked to the pathophysiology of many diseases (Juster et al., 2010). The most studied of all stress related disorders is cardiovascular disease (CVD), which has been highlighted as the leading cause of mortality worldwide (World Health Organization, 2011). Previously, stress experienced by a person at their place of employment or work has been suggested to substantially increase their CVD risk (Yarnell, 2008; Thayer et al., 2010) with the risk of coronary heart disease being increased by 50% among workers (Kivimaki et al., 2006). As individuals spend most of their daily time at work (e.g., 8–10 h per day), a greater examination of the impact of interventions focusing on managing work-related stress as an important component of allostatic loads is warranted to reduce CVD risk and promote life-long resiliency against abnormal allostatic loading for workers (Juster et al., 2010). Development of healthy workplaces and practices may provide important environments that combat chronic stress and its consequent adverse contribution to the increasing work-related development of disease (Taylor et al., 1997).

Physical activity (PA) and exercise have been extensively recognized as important influences on the relationship between psychosocial stress and CVD (Hamer, 2012) probably because of its influence on physical fitness. Thus, it would be expected that more active individuals, who conversely possess higher physical fitness, would be more resilient to mental stresses (Hamer, 2012). In this regard, interventions that involve PA in conjunction with other beneficial practices (e.g., social support) in the workplace may be very effective for the control of allostatic load at an individual level (Juster et al., 2010). However, the expected greater stress resilience in those individuals with a greater physical fitness has been questioned as the stress-buffering effect of physical fitness has not always been demonstrated (Jackson and Dishman, 2006). This lack of demonstration may be related to methodological constraints of previous studies (Hamer, 2012) with further studies warranted to elucidate the important role that PA and exercise could have on workers cardiovascular health, potentially as an important stress-buffer.

Heart rate variability (HRV) is an easy and non-invasive tool for the assessment of variations in beat-to-beat intervals and autonomic nervous system activity with HRV obtained by linear methods within the domains of time and frequency analyses, and nonlinear methods (Task Force, 1996). HRV has been studied extensively in regards to CVD (Vrijkotte et al., 2000; Kivimaki et al., 2006; Yarnell, 2008; Thayer et al., 2010; Frodl and O'Keane, 2012), exercise (Proper et al., 2002; Kiviniemi et al., 2007; Buchheit et al., 2010; Boullosa et al., 2012), and stress (Hjortskov et al., 2004; Collins et al., 2005; Yarnell, 2008; Loerbroks et al., 2010; Uusitalo et al., 2011). The use of HRV as a practical monitoring tool for allostatic load though has been scarce and may provide a simple instrument for workers in the workplace. Greater HRV has been related to lower cardiovascular risk (Kiviniemi et al., 2010), greater physical fitness and responsiveness to aerobic training (Hautala et al., 2010), greater PA levels in workers (Rennie et al., 2003), and lower work related stress in workers (Uusitalo et al., 2011). Collectively, these and other previous studies (Orsila et al., 2008; Thayer and Lane, 2009; Hynynen et al., 2011) emphasize the importance of HRV for the assessment of cardiovascular stress in the workplace with PA and fitness potentially enhancing HRV control. Therefore, the aim of this mini-review was to review the current literature regarding HRV, PA, fitness, and workplace stress to better delineate the current understanding and potential for future studies.

## Materials and methods

**Figure 1:**
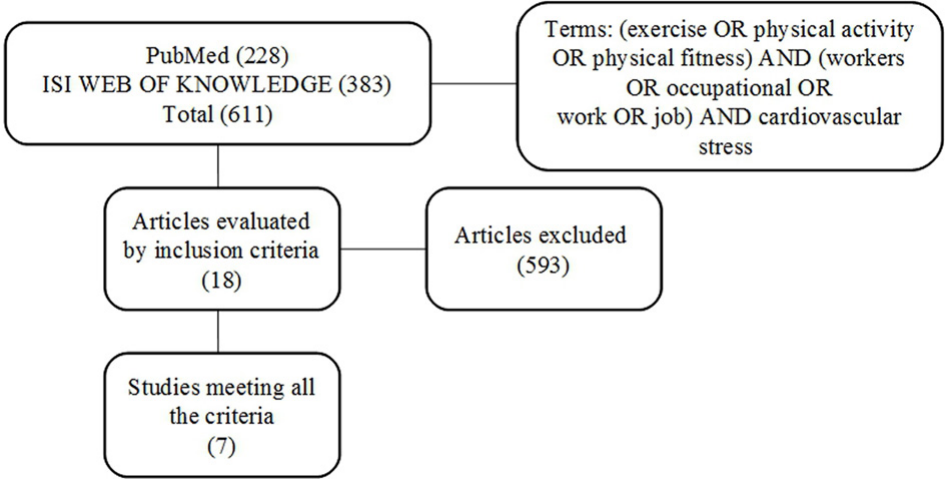
**Overview of search strategy**.

An extensive search of relevant studies listed within the National Library of Medicine (PubMed) and ISI Web of Knowledge databases over the past 10 years (2003–2013) was conducted. The following combination of terms was utilized during the search: (exercise OR PA OR physical fitness) AND (workers OR occupational OR work OR job) AND cardiovascular stress (Figure 1). Inclusion criteria included: original articles; written in English; study population consisted of workers; included assessments of PA and/or exercise; assessment of work-related stress, and cardiac autonomic control monitored via HRV. The review, evaluation and selection of articles based upon inclusion criteria were carried out independently by three of the authors.

## Results

After initial searches based on the combination of specific terms were performed, there were 228 and 383 articles identified within PubMed and ISI Web of Knowledge databases, respectively (Figure 1). The titles and abstracts of these 611 articles were reviewed with 593 excluded as not meeting the inclusion criteria. Subsequently, 18 articles were obtained in full and examined further for details according to the inclusion criteria (Figure 1). Based upon these criteria, only seven articles were included in this study (see Table 1). These studies examined autonomic function via HRV for job stress evaluation, however studies included a variety of analyses, different scheduling of R-R recordings, and various populations that make comparisons difficult. Nevertheless, several studies (Chandola et al., 2008; Clays et al., 2011; Uusitalo et al., 2011) reported a reduced HRV in workers reporting work-related stress. Interestingly, only Chandola et al. (2008) reported a positive association between work stress and CVD possibly as a consequence of the negative influence of stress on health behaviors (i.e., reduced PA and poor diet). Meanwhile, others (Eller et al., 2011b; Lindholm et al., 2012) did not find any direct relationship between stress and HRV indices although a reduced HRV was exhibited during an imbalance between effort and reward, and in those workers experiencing irregular shiftwork, important sources of work-related stress. Likewise Melville et al. (2012) and Cheema et al. (2013) reported no relationship between HRV and stress with reductions in stress and anxiety following workplace yoga interventions accompanied by no changes in HRV.

**Table 1:** **Summary of studies involving HRV, work stress, and physical activity/exercise in the last 10 years**.

**Authors**	**Characteristics of participants**	**Assessment of stress**	**Assessment of physical activity/exercise**	**Evaluation method of HRV**	**Results**
Chandola et al., 2008	A total of 10,308 London-based male and female civil servants aged 35–55 years	Self-reported work stress was measured by the job-strain questionnaire	Physical activity was measured by self-reported frequency of moderate activities (e.g., three times a week, at least once a week, at least once a month, never)	HRV—5 min of RR interval data were collected	There was an association between work stress and low HRV for participants at all ages. Greater reports of work stress were associated with lower HRV (LF, HF, and TP). Around 32% of the effect of work stress on Coronary Heart Disease (CHD) was explained by the effect of work stress on health behaviors (i.e., low physical activity and poor diet in particular) and the metabolic syndrome. The association between work stress and CHD was stronger among employees younger than 50 and those still in employment
Uusitalo et al., 2011	19 adults (18 women and 1 man), average 42 years (range 24–57 years)	Effort-reward imbalance (ERI) questionnaire: Effort refers to the demanding aspects of the work environment (six items, rating scale from 1 to 5): Reward refers to esteem, career opportunities and job security (11 items, rating scale from 1 to 5) A score of imbalance was obtained by calculating the ratio between Effort and Reward	*Actiwatch activity monitoring system* (Neurotechnology Ltd., Cambridge, UK).	Two 36-h, measurements were recorded on two different work days—36–84-h. Data were measured with Polar RR- recorder during work and at home	No significant differences were identified between daytime and work time physical activity scores. Daytime HRV (i.e., RMSSD) correlated significantly and negatively with daytime stress feelings on both days. Work time irritation correlated negatively with night time HRV (i.e., SDNN, RMSSD, and LF) on both days. The relationship between worker physical activity level and HRV, and stress at work, was not examined
Clays et al., 2011	653 healthy male workers. (40–55 years)	Job Stress Questionnaire (JSQ) contained 27 questions, were reduced and treated as 18 separate items for a Total JSQ score.	The Minnesota Leisure Time Physical Activity questionnaire: assessment of mean energy expenditure during leisure time physical activity in the past 12 months	Mean 24-h of ambulatory ECG recordings. HRV assessed during regular activities on a working day	Leisure time physical activity score median (IQR) = 70.05 (37.75–1.14). The relationship between worker physical activity level and HRV, and stress at work, was not examined. Both the JSQ scale and the adapted Work Stressor Index were positively and significantly related to mean HR and HRV (i.e., LF/HF). A significant negative correlation was reported between the Work Stressor Index and HRV (i.e., pNN50 and HF)
		Additionally, an adapted scale based on only five items: general satisfactions at work, responsibilities at work, imposed work pace, difficult professional relations and complaints about physical work conditions were examined as a Work Stressor Index			
Eller et al., 2011a	231 public sector workers (Mean age of 49.3 ± 8.8 for females, 51.2 ± 9.7 for males)	Effort-reward imbalance (ERI) questionnaire. Effort was evaluated by four questions and reward was evaluated by seven questions; answers were provided using a five point scale. Score of imbalance was obtained by calculating the effort/(reward × 4/7)	The degree of physical activity was measured via questionnaire, using four levels from very low activity: 1 = almost physical passive to high activity: 4 = intensive physical activity for more than 4 h/week	Ambulatory electrocardiograms (~18 h) Commencing between 9:00–12:00 at the work place and ending the next morning	45.5% of women and 34.4% of men were engaged in physical activity for 2–4-h per week. Consistent associations between the psychosocial work environment (i.e., ERI-model) and HRV. The ranges of the ERI were higher for women (3.5) when compared to men (2.5). Analyses including ERI, were adjusted for sex, year, age, and time of measurement showed that women had significantly higher lnHR and lower ln(LF/HF) compared with men
Lindholm et al., 2012	66 workers with irregular shift work (ISW) and 66 workers with normal daytime work (RDW) (age 41.3 ± 10.3)	Questionnaire with several items: demographics, general health experience, physical health status, sleep, and insomnia symptoms, psychosocial status, stress, work satisfaction, and performance (Ahlberg et al., 2008)	Questionnaire with several items including leisure time exercise	Measured by ambulatory long-term ECG (24 h)	Regular weekly physical activity (leisure time exercise, walking/cycling to work) was reported by 84% of workers. Approximately 69% of ISW workers and 60% of RDW workers undertook less than three sessions of physical activity per week. The ISW group exhibited lower values of RMSSD in the late evening and first hours of sleep with insufficient recovery from daytime sleepiness.
Melville et al., 2012	20 sedentary workers aged 39.6 ± 9.5 years (8 women)	Means of a 100 mm visual analog scale in which participants rated their state of stress/relaxation at that particular moment, ranging from “extreme relaxation” (0 mm) to “extreme stress” (100 mm)	Three acute sessions were examined: 15 min of yoga, 15 min of meditation, and 15 min of no exercise (control) with each session separated by ≥24-h. Each session was followed by 15 min of recovery	Short-term recordings of heart rate and HRV continuously using a telemetric monitor. Data were grouped into seven 5-min phases including 1 × 5 min recording at baseline, 3 × 5 min recordings during and following each session	Compared to the control session, HR was significantly greater during yoga (6.5%) and significantly lower during meditation (3.9%) with HR during the yoga and meditation sessions significantly different. HRV (SDNN and TP) were significantly reduced during the yoga and meditation sessions compared to control. Perceived stress was significantly decreased immediately after the yoga (*p* < 0.003) and meditation (*p* < 0.000) sessions vs. control, and these effects were maintained for 15 min during recovery.
Cheema et al., 2013	Academic and general staff of a university divided into yoga (*n* = 18; 10 weeks hatha yoga sessions, three times a week, during lunch break, 50 min per session), and control (*n* = 19) groups	Job Descriptive Index (JDI); Job in General (JIG) scale	Upper-body muscular endurance was evaluated using a standardized push-up test. Low-back and abdominal endurance was evaluated by means of an isometric, side-bridge test. Low-back and hip flexibility was evaluated via standardized sit-and-reach test	10 min ECG recordings before (Week 0) and after the intervention (Week 10, at least 48 h following the final yoga session in those randomized to the experimental group)	Log HF was not significantly improved in the yoga group vs. the control group over time (*p* = 0.48). The yoga group significantly reduced pNN50 (*p* = 0.04) and increased log LF/HF (*p* = 0.04) vs. the control group. The yoga intervention significantly increased low-back and hip flexibility (*p* < 0.001). *Post-hoc* analysis comparing participants who completed ≥70% of yoga sessions (*n* = 11) to control (*n* = 19) yielded the same findings, except that the high adherers also reduced state anxiety (*p* = 0.02) and RMSSD (*p* = 0.05), and tended to improve the push-up test (*p* = 0.07) vs. control.

Abbreviations: RMSSD, root mean squared differences of successive NN intervals; pNN50, % differences between adjacent RR intervals N50 ms; SDNN, standard deviation of normal-to-normal (NN) intervals; CHD, Coronary Heart Disease; LF, Low Frequency; LF/HF, Low Frequency to High Frequency Ratio; HR, Heart Rate; HF, High Frequency; JSQ, Job Stress Questionnaire; ISW, Irregular shift work; RDW, Regular daytime work; ERI, Effort-reward imbalance; IQR, interquartile range. HRV, heart rate variability; ECG, electro cardiogram; lnHR, normalized heart rate; ln(LF/HF), normalized Low Frequency to High Frequency Ratio; TP, total power; JDI, Job Descriptive Index; JIG, Job in General scale.

All included studies assessed the level of PA or exercise with most simply reporting the level in a descriptive function. Only one study (Cheema et al., 2013) examined PA or exercise as an independent variable in a longitudinal design of work related stress and autonomic control of HR. Surprisingly, this recent study (Cheema et al., 2013) reported a worsening of autonomic control of HR (i.e., decrements in HRV indices) for the yoga intervention group vs. the control group. Paradoxically, the participants of this study exhibited improvements in some fitness characteristics and a reduced anxiety state after the yoga intervention suggesting an effective intervention but with an unexpected reduced HRV (Cheema et al., 2013). Previously, Melville et al. (2012) examined the acute effects of both yoga and meditation interventions on perceived stress and HRV with no significant changes in HRV during the acute post-intervention period (i.e., 15 min), despite significant reductions in perceived stress after both interventions.

## Discussion

This review suggests that autonomic function could be a simple and effective measure to identify workplace stress. Additionally, a single study with longitudinal workplace PA interventions reported improved worker stress levels without significant positive changes in cardiac autonomic activity, a mechanism known to be cardioprotective. Based upon the current few studies of PA and stress, and equivocal relationships between stress and cardiovascular health in workers, we encourage more studies involving varying PA and exercise interventions in the workplace to better examine the benefits of PA and exercise on both stress and HRV.

Overall, the current review provides further support of the applicability of cardiac autonomic function monitoring for work related stress. Factors related to adverse working conditions such as excessive effort (Vrijkotte et al., 2004), effort-reward imbalance (Eller et al., 2011a; Uusitalo et al., 2011), over commitment (Vrijkotte et al., 2004; Lindholm et al., 2012), irregular shift work (Lindholm et al., 2012), and work stress (Chandola et al., 2008) were significantly related to reduced cardiac autonomic function. Therefore, HRV monitoring may provide a simple and non-invasive assessment of stress and allostatic load in working environments that employers could utilize in the efficient management of employees. The recent systematic review of Jarczok et al. (2013) provides further support for HRV use in employee management with adverse psychosocial work conditions reported to be negatively associated with autonomic nervous system function as indexed by HRV.

It should be pointed out that within the current review, a variety of different HRV analyses were employed that limited comparisons between studies. The selection of HRV methodology is an important issue as tool sensitivity may compromise the detection of cardiac autonomic responses (Hynynen et al., 2011). Aspects such as variations in HRV measures examined (e.g., linear, non-linear, etc.), data analysis (e.g., supine, seated, Fast Fourier Transform, Autoregression, etc.) as well as the quality of data (e.g., degree of ectopy/artifact, sampling rate, recording length, etc.) make comparisons between studies challenging (Jarczok et al., 2013). For instance, Hynynen et al. (2011) reported that HRV during an orthostatic test upon wakening may be more useful for the analysis of stress in real life compared to night time recordings. In contrast, HRV measures recorded during both work and at night were reported to be also sensitive markers of mental stress alterations at work (Hjortskov et al., 2004; Yarnell, 2008). Further studies should determine the optimal HRV methods for detecting cardiac autonomic stress related adaptations. Factors such as the HRV measure (i.e., time or frequency domain, nonlinear, etc.), time, and duration of recordings and body posture (Young and Leicht, 2011; Boullosa et al., 2012) may play an important role that requires further clarification. Given the possible influence of factors external to work related stressors during long recordings, the use of laboratory controlled recordings should be included in further studies for a better evaluation of autonomic control of HR (Lombardi and Stein, 2011). Additionally, other cardiac autonomic indices (i.e., heart rate recovery; HRR, post-exercise) could also be employed as a delayed HRR was reported in individuals with high levels of stress and depression (Gordon et al., 2012). Consequently, the simple assessment of HRV and HRR may provide a comprehensive evaluation of the cardiac autonomic health of workers and prognosis for work-related stress and responsiveness to exercise training (Huovinen et al., 2011).

Another important restriction of studies within the current review was the different assessment tools for stress levels or psychosocial characteristics of work. For instance, the following tools were utilized in the current review studies: effort reward imbalance model (ERI-model) (Eller et al., 2011a; Uusitalo et al., 2011), Job Descriptive Index (JDI); Job in General (JIG) scale (Cheema et al., 2013), job stress questionnaire (Clays et al., 2011), stress questionnaire (Lindholm et al., 2012), job-strain questionnaire (Chandola et al., 2008), and a 100 mm visual analog scale (Melville et al., 2012). While each of the aforementioned tools has been used to assess stress at work, differences in specificity, reliability, and validity of these tools and their relationship with cardiac autonomic activity made comparisons difficult. Standardized use of stress assessment tools may help clarify the relationship between stress and cardiac autonomic activity. For example, Jarczok et al. (2013) highlighted that measurements of stress could be grouped when evaluated by ERI and JDC questionnaires with these questionnaires demonstrating a significant association between high strain/stress work and decreased vagal tone. Identification of the ultimate assessment tool may help to better understand the relationship between cardiovascular stress and workers health. Further, this tool may also overcome the confounding variables that affect the measurement of cardiac autonomic control such as age, sex, disease, caffeine intake, PA, smoking, and alcohol consumption (Jarczok et al., 2013).

One disappointing aspect of the current review was the low incidence of exercise and PA with only two studies involving exercise interventions (i.e., yoga or yoga and meditation) (Melville et al., 2012; Cheema et al., 2013) in the workplace. Further, these recent studies (Melville et al., 2012; Cheema et al., 2013) revealed contradictory results regarding the relationship between exercise interventions, stress, and HRV. The remaining studies (Vrijkotte et al., 2004; Clays et al., 2011; Eller et al., 2011a; Uusitalo et al., 2011; Lindholm et al., 2012) simply recorded PA as a factor of consideration in their analyses. Moreover, only one study objectively recorded the levels of PA via accelerometry (Uusitalo et al., 2011) with the remaining studies utilizing questionnaires for this purpose. Further, very few examined physical fitness with those studies assessing physical fitness (Clays et al., 2011; Uusitalo et al., 2011; Cheema et al., 2013) and not the potential role of this factor on stress responses and HRV. Therefore, the results of our review confirmed the variety of factors utilized in past studies with future studies recommended to examine objective measures of exercise and PA as these have been previously associated with HRV levels (Rennie et al., 2003; Hautala et al., 2010; Takada et al., 2010).

Previous studies have observed a weak and inverse association between job strain and leisure time PA indicating the potentially important role of PA for managing work related stress (Loerbroks et al., 2010). Previously, Choi et al. (2010) reported that having on-the-job learning opportunities and decision authority about their tasks may be conducive to increasing active leisure time PA in middle-aged US workers. While these previous studies have analyzed the impact of work conditions on leisure time PA, other studies have analyzed the impact of complex interventions including exercise in the workplace. For instance, Eriksen et al. (2002) previously demonstrated the greater effectiveness of complex interventions including PA than more focused non-PA interventions (e.g., stress management alone). Subsequently, Tveito and Eriksen (2009) reported in a randomized controlled study that a program consisting of physical exercise, stress management training, health information and an examination of the participants' workplace, promoted improvements in health, physical fitness, muscle pain, stress management, maintenance of health, and work situation. Overall, these previous studies highlighted the appropriateness of complex, exercise or PA based interventions at work for stress management and other health related outcomes. Paradoxically, in our focused review we found only two studies with workplace exercise interventions. Melville et al. (2012) evaluated the impact of a brief intervention (e.g., 15 min of yoga) at work on the acute cardiovascular responses (i.e., HR, HRV, and blood pressure) and perceived stress. Interestingly, this study reported a higher HRV during yoga and meditation interventions that returned to baseline immediately after the session with a reduction of perceived stress for 15 min post-intervention (Melville et al., 2012). Although the results of this study were promising, these results, within a small time of evaluation, should be considered with caution, especially due to the low intensity and duration of yoga practice. However, such an exercise intervention could be promising for the control of acute work-related stress responses (Hamer, 2012). Another study (Cheema et al., 2013) also evaluated the effectiveness of a workplace program based of hatha yoga (10 weeks, 50 min at lunch time, 3 times per week) on physiological stress (HRV) and associated health-related outcomes in a cohort of office workers. Despite the reduction of anxiety and fitness improvements among those with high adherence rates, the decrease in HRV after the intervention period raised concerns about the effectiveness of this exercise modality for the cardiac health of workers. Further studies with other exercise and PA interventions, which are different concepts (Caspersen et al., 1985), may clarify the most appropriate modality and dose-response for the control of stress and associated CVD in workers.

In the current review, most studies were cross-sectional with only two involving exercise interventions (yoga and meditation). Based on this review, there is a notable lack of studies comparing longitudinal interventions and the cause-effect relationships between physical fitness, exercise, and PA, and cardiovascular stress (Hamer, 2012). Moreover, identification of barriers and facilitators for PA or exercise at work (Renton et al., 2011; Leicht et al., 2013) may assist with the development of the most appropriate interventions to assist workers based on their employment demands. For instance, emergency physicians with different shifts (14 or 24-h) exhibited significant differences in stress levels (Dutheil et al., 2012). Such factors may assist in developing the best interventions and any potential changes in working environments (Conn et al., 2009). Although the use of complex interventions including exercise, psychological and nutritional habits have been previously recommended (Carson et al., 2010; Strijk et al., 2012), the inclusion of sole exercise and/or PA interventions may determine the appropriate dose-response (Vanhees et al., 2012). This would be very important as the levels of physical fitness that protect against CVD when considering specific job demands remain undefined (Huang and Acevedo, 2011). Moreover, CVD risk factors and stress exhibit a bidirectional relationship as stress promotes changes in behavior and pathophysiological parameters and vice versa (Huang and Acevedo, 2011) that should be considered in both prevention and treatment interventions (Stults-Kolehmainen, 2013). Thus, stressed workers may be less inclined to perform PA contributing to a greater deterioration of their health and progression of pathophysiological conditions (Azevedo et al., 2012; Silva and Barreto, 2012). Identification of barriers and motivators within workers would be an important aspect for designing effective and individualized intervention programs that also considers PA levels and sitting time derived from specific job activities (Leicht et al., 2013).

In summary, the results of the current review suggested a relationship between work related stress and autonomic control of HR. However, the extent of the positive benefits of both PA and exercise on both stress and HRV remains to be elucidated. Thus, future research, especially longitudinal studies, with different work categories and samples are needed to better understand how different levels of physical fitness, energy expenditure and exercise modes prevent or minimize the allostatic load and subsequent stress on workers and their impact on cardiovascular health.

### Conflict of interest statement

The authors declare that the research was conducted in the absence of any commercial or financial relationships that could be construed as a potential conflict of interest.
